# Lipopolysaccharide Downregulates CD163 Expression to Inhibit PRRSV Infection via TLR4-NF-κB Pathway

**DOI:** 10.3389/fmicb.2020.00501

**Published:** 2020-03-25

**Authors:** Zhenbang Zhu, Hui Zhang, Xiaoxiao Zhang, Sheng He, Wenjuan Dong, Xiaoying Wang, Yaosheng Chen, Xiaohong Liu, Chunhe Guo

**Affiliations:** State Key Laboratory of Biocontrol, School of Life Sciences, Sun Yat-sen University, Guangzhou, China

**Keywords:** PRRSV, lipopolysaccharide, proinflammatory response, ADAM17, CD163

## Abstract

Porcine reproductive and respiratory syndrome virus (PRRSV) has been recognized to induce proinflammatory cytokine production and modulate the host interferon (IFN) system. Proinflammatory cytokines and type I IFNs contribute to the prevention of viral infection. Lipopolysaccharide (LPS), a specific agonist to Toll-like receptor 4 (TLR4), provokes signal transduction and activates immune response *in vivo* and *in vitro*. Here we identified LPS inhibited PRRSV infection in porcine alveolar macrophages (PAMs) and in Marc-145 cells. To investigate the possible mechanism, we found TLR4-NF-κB pathway was obviously activated in LPS-treated PAMs at the early stage of PRRSV infection. As a result, the expression of proinflammatory cytokines was strongly induced following LPS and PRRSV co-treatment. Due to the enhanced proinflammatory response, CD163 expression was significantly reduced and a disintegrin and metalloproteinase 17 was activated, which promotes the cleavage of membrane CD163. Ultimately, CD163 down-regulation led to the suppression of PRRSV replication. Our data demonstrate that LPS has an impact on PRRSV infection via inflammation response, which provides a new insight of inflammation-mediated antiviral immunity and a new strategy to control PRRSV infection.

## Introduction

Porcine reproductive and respiratory syndrome (PRRS), caused by PRRS virus (PRRSV), is a detrimental infectious swine disease. The main clinical symptoms of infected pigs appear severe reproductive failure in pregnant sows and respiratory disorder in piglets and growing pigs (Cho and Dee, 2006). Since its emergence in the late 1980s, the disease has been accompanying with the global swine production, which also brings considerable economic losses to the world’s swine industry every year (Lunney et al., 2010). PRRSV is a single-stranded, positive-sense RNA virus and belongs to the genus Porartevirus of the family Arteriviridae of the order Nidovirales. The genome size of PRRSV is approximately 15.4 kb, containing at least 11 open reading frames (ORFs) (Dokland, 2010). PRRSV non-structural proteins and structural proteins are encoded by these ORFs, which participate in pathogenesis of infection (Music and Gagnon, 2010). PRRSV is characterized by high variation, persistent infection and antibody-dependent enhancement, so that PRRS has not been effectively controlled until now. Specific receptors on cell surface are responsible for PRRSV infection. The related receptors mediating PRRSV infection include vimentin, heparin sulfate, CD151, sialoadhesin (CD169), DC-SIGN (CD209), and the most indispensable receptor CD163 (Van Breedam et al., 2010).

CD163 is a key factor for PRRSV infection via promoting viral uncoating. CD163 is expressed in some non-permissive cells exogenously, which renders them permissive for PRRSV infection (Wang et al., 2013). Different CD163 gene edited pigs (CD163-null pigs, CD163 domain swapping pigs and CD163 SRCR5 deletion pigs) were generated and had the protection against both PRRSV 1 and 2 in recent years (Whitworth et al., 2016; Guo et al., 2019). Therefore, CD163 plays a pivotal role in PRRSV infection, the level of CD163 expression determines the viral load of PRRSV infection. CD163 expression can be regulated by a variety of factors *in vitro*. Expression of CD163 is strongly induced by glucocorticoids and interleukin (IL)-10. Inversely, CD163 expression is down-regulated by proinflammatory cytokines including IL-1α, IL-1β, IL-4, IL-8, tumor necrosis factor-alpha (TNF-α), and even lipopolysaccharide (LPS). Activation of Toll-like receptor 2 (TLR2), TLR4, or TLR5 results in a reduction of CD163 expression (Van Gorp et al., 2010; Kowal et al., 2011). This down-regulation of CD163 expression is mediated by a disintegrin and metalloproteinase 17 (ADAM17) (Etzerodt et al., 2010; Etzerodt and Moestrup, 2013). Therefore, CD163 is involved in inflammation and regulated by pro- and anti-inflammatory mediators.

PRRSV replication triggers the activation of NF-κB, ERK1/2, and p38MAPKs, which results in the release of proinflammatory cytokines such as IL-1β, IL-8, IL-6, and TNF-α (Zhou and Zhang, 2012; Gomez-Laguna et al., 2013). In parallel, NF-κB, ERK1/2, and p38MAPKs are downstream signaling of TLR4 receptor. PRRSV increased IL-1β secretion via TLR4/MyD88 pathway and downstream signaling molecules (Bi et al., 2014). LPS, a component of the outer membrane of Gram-negative bacteria, binds myeloid differentiation factor 2 (MD2) and TLR4, forming TLR4-MD2-LPS complex (da Silva Correia et al., 2001; Schromm et al., 2001; Akashi et al., 2003). TLR4 responds to bacterial LPS. TLR4-MD2-LPS complex initiates signal transduction of IRF3 and NF-κB and consequently leads to secretion of type I interferon and inflammatory cytokines (Kawai and Akira, 2010). Moreover, LPS-stimulated PAMs polarizes toward M1 PAMs, which significantly inhibits PRRSV replication, but it remains an unclear mechanism (Wang et al., 2017).

We investigated whether LPS had an impact on PRRSV infection via proinflammatory response. In this study, we found that LPS stimulation significantly decreased PRRSV infection *in vitro*. LPS accelerated the activation of TLR4-NF-κB pathway and potentiated the release of proinflammatory cytokines during PRRSV infection. The strong induction of proinflammatory cytokines leads to the increase of ADAM17 expression and down-regulation of CD163 expression, which results in suppression of PRRSV replication. Thus, LPS could regulate PRRSV infection via inflammation response. Our findings provide new insights to the underlying mechanism that LPS restrains PRRSV infection and proinflammatory cytokines play important regulating roles in antiviral immune response.

## Materials and Methods

### Ethics Statement

Most of our experiments were performed on porcine alveolar macrophages (PAMs). PAMs were isolated from bronchoalveolar lavage fluid (BALF) of three 6-week-old piglets. All animal experiments were approved by the Institutional Animal Care and Use Committee of Sun Yat-sen University. All animal work was carried out under the Laboratory Animals – Guideline of welfare and ethics written by the General Administration of Quality Supervision, Inspection and Quarantine of the People’s Republic of China.

### Cells and Viruses

PAMs were isolated from BALF of 6-week-old piglets. Three pigs were euthanatized and we removed the lungs from thoracic cavity. Phosphate buffer solution (PBS, Corning, United States) were used to clean the surface of lungs and lavage into the lungs for three times. BALF were centrifuged for 10 min at 129 × g and cells were obtained. After removing the supernatants, cells were resuspended with RPMI 1640 medium (Gibco, United States) and collected by centrifugation for 5 min at 129 × g. Finally, cells were frozen in 40% RPMI-1640 medium, 50% fetal bovine serum (FBS) (PAN, Germany), and 10% DMSO (Sigma, United States). PAMs were cultured in RPMI 1640 medium with 10% FBS at 37°C in 5% CO_2_. No antibiotic was used in the cell culture during the experiments. In PAMs isolation, all piglets were full sibs and from a litter of pigs. Three paralleled repetitions were included for each treatment. The reproducibility of the results among different PAMs batches were well performed. Marc-145 cells (China Center for Type Culture Collection, China), derived from African green monkey embryonic kidney cells, an immortalized cell line which were commonly used for the research of PRRSV infection, were cultured in Dulbecco’s modified Eagle’s medium (DMEM) (Corning, United States) containing 10% FBS at 37°C in 5% CO_2_. CHR6 is classified as classical North American type PRRSV strain, which was provided by Dr. Heng Wang from South China Agricultural University. CHR6 strain was propagated in Marc-145 cells and titrated as 50% tissue culture infective dose (TCID_50_).

### LPS Treatment

PAMs were seeded in six-well plates (2 × 10^6^/well). When cells adhered to cell-culture dish, cells were mock treated or treated with different concentrations of LPS (0, 100, or 200 ng/mL) for 6 h, following the supplement with fresh RPMI 1640 medium containing 2% FBS. After LPS stimulation, cells were rinsed with PBS for three times and then mock infected or infected with CHR6 at indicated mutiplicity of infection (MOI) for indicated time points. Cells were harvested for western blot, qRT-PCR, immunofluorescence and flow cytometry analysis. Three replicates were included for each treatment.

### Detection of Proinflammatory Cytokines

PAMs were mock stimulated or stimulated with LPS (100 ng/mL) for 6 h, and then infected with CHR6 (MOI = 1) for indicated time points (0, 3, 6, and 12 h). To assess the effect of LPS on transcription of proinflammatory cytokines in PRRSV infected cells, relative expressions of IL-1β, IL-6, IL-8, and TNF-α were detected using qRT-PCR and calculated using the 2^–Δ^ ^Δ^ ^*Ct*^ method. Three replicates were included for each treatment.

### Western Blot

Six-well-plate cell samples (2 × 10^6^/well) were harvested and lysed in a cell lysis buffer containing a protease inhibitor cocktail and phosphatase inhibitor cocktail. The total protein for each sample was measured with a BCA protein assay kit (Thermo Scientific, United States). Whole cell lysates were subjected to 12% sodium dodecyl sulfate-polyacrylamide gel electrophoresis (SDS-PAGE) and transferred to polyvinyl difluoride (PVDF) membrane (Roche, United States). Membranes were blocked with 3% BSA (Ruishu, China) in TBST (20 mM Tris-HCl PH8.0, 150 mM NaCl, 0.05% Tween 20) at room temperature for 1 h, and then incubated with the indicated primary antibodies diluted in TBST buffer containing 3% BSA at 4°C overnight. After washing four times with TBST buffer, membranes were incubated with indicated secondary antibodies diluted in TBST buffer containing 3% BSA. GAPDH serves as an internal control. Protein signals were visualized using a chemiluminescence (ECL) reagent (Fdbio Science, China). Three replicates were included for each treatment. Primary antibodies were used as follows: anti-PRRSV N (4A5) antibody (9041) was purchased from MEDIAN Diagnostics (MEDIAN, Republic of Korea); anti-ADAM17 antibody (ab39163), anti-CD163 antibody (ab87099) were purchased from abcam (abcam, England); GAPDH (14C10) Rabbit mAb (2118), Phospho-Akt (Ser473) (D9E) XP^®^ Rabbit mAb (4060S), Akt Antibody (9272), p38 MAPK (D13E1) XP^®^ Rabbit mAb (8690), Phospho-p38 MAPK (Thr180/Tyr182) (D3F9) XP^®^ Rabbit mAb (4511), p44/42 MAPK (Erk1/2) (137F5) Rabbit mAb (4695), Phospho-p44/42 MAPK (Erk1/2) (Thr202/Tyr204) (D13.14.4E) XP^®^ Rabbit mAb (4370), NF-κB p65 (D14E12) XP^®^ Rabbit mAb (8242), Phospho-NF-κB p65 (Ser536) (93H1) Rabbit mAb (3033), Myc-tag (9B11) mouse mAb (2276) were purchased from Cell Signaling Technology (CST, United States).

### Quantitative Real-Time Reverse-Transcription Polymerase Chain Reaction (qRT-PCR)

The mRNA expression of PRRSV N, ADAM17, CD163, TLR4, NF-κB, and various proinflammatory cytokines (IL-1β, IL-6, IL-8, and TNF-α) was assessed by qRT-PCR. PAMs (2 × 10^6^/well) were washed three times with PBS and total RNA were extracted from cells using the TRIzol reagent. RNA were reverse-transcribed into cDNA using Reverse Transcription System (A3500, Promega, United States) according to manufacturer’s instructions. Reverse-transcription products were amplified with indicated primers using 2 × RealStar Green Power Mixture (GenStar, China). The primers used for qRT-PCR are listed in Table 1. Real-time PCR was performed on a LightCycler 480 Real-Time PCR System (LC480, Roche, Switzerland). Data were normalized to HPRT1 or GAPDH in each individual sample. Relative mRNA expression was calculated using the 2^–ΔΔ*Ct*^ method. Each assay was performed in triplicate. Three replicates were included for each treatment.

**TABLE 1:** List of primers for qRT-PCR.

**Primer^a^**	**Sequence (5′–3′)^b^**
ORF7 (N)-F	AAAACCAGTCCAGAGGCAAG
ORF7 (N)-R	CGGATCAGACGCACAGTATG
ADAM17-F	GCACAGGTAATAGCAGTGAGTGC
ADAM17-R	CACACAATGGACAAGAATGCTG
CD163-F	ATTCATCATCCTCGGACCCAT
CD163-R	CCCAGCACAACGACCACCT
IL-1β-F	CCCAAAAGTTACCCGAAGAGG
IL-1β-R	TCTGCTTGAGAGGTGCTGATG
IL-6-F	CTGCTTCTGGTGATGGCTACTG
IL-6-R	GGCATCACCTTTGGCATCTT
IL-8-F	AGTTTTCCTGCTTTCTGCAGCT
IL-8-R	TGGCATCGAAGTTCTGCACT
TNF-α-F	ACTCGGAACCTCATGGACAG
TNF-α-R	AGGGGTGAGTCAGTGTGACC
TLR4-F	CCTGCCTGTGCTGAGTTTCA
TLR4-R	AAGGTGAGAACTGACGCACTAATG
NF-κB-F	TCGCTGCCAAAGAAGGACAT
NF-κB-R	AGCGTTCAGACCTTCACCGT
HPRT1-F	TGGAAAGAATGTCTTGATTGTTGAAG
HPRT1-R	ATCTTTGGATTATGCTGCTTGACC
mGAPDH-F	TGACAACAGCCTCAAGATCG
mGAPDH-R	GTCTTCTGGGTGGCAGTGAT

**^*a*^F, forward primer; R, reverse primer. ^*b*^Pig gene sequences and PRRSV gene sequences were downloaded from GenBank.**

### Immunofluorescence

Cells (1 × 10^6^/well) growing on twelve-well plates were fixed with paraformaldehyde (Ruishu, China) for 10 min and then permeabilized by PBS containing 0.5% Triton X-100 (Ruishu, China) for 15 min at the room temperature. After rinsing with PBS for three times, cells were blocked with 1% BSA for 1 h at the room temperature. Then, samples were incubated with the indicated primary antibodies for overnight at 4°C. After incubating with the indicated secondary antibodies for 1 h, cells were counterstained with DAPI in PBS for an additional 5 min. All images were captured and processed using an inverted fluorescence microscope (NIKON ECLIPSE Ti2-U, Japan) and a confocal laser scanning microscope (TCS-SP5, LEICA, Germany). Three replicates were included for each treatment. Primary antibodies were used as follows: anti-PRRSV N (4A5) antibody (9041) (1:500 dilution, MEDIAN, Republic of Korea); anti-ADAM17 antibody (ab39163) (1:500 dilution, abcam, England); anti-CD163 antibody (ab87099) (1:500 dilution, abcam, England); NF-κB p65 (D14E12) XP^®^ Rabbit mAb (8242) (1:500 dilution, CST, United States).

### Flow Cytometry

We used flow cytometry to detect the expression of membrane CD163. PAMs were mock treated or treated with LPS for 6 h, and then infected with CHR6 for 24 h. Cells were gently rinsed with PBS for three times, dissociated from the plates with 0.25% Trypsin at 37°C for 2 min. PAMs were fixed with 4% paraformaldehyde for 10 min, followed by three times wash with PBS. Cells were stained with anti-pig CD163-FITC monoclonal antibody (1:500 dilution, Bio-Rad, United States) diluted in PBS containing 1% BSA for 1 h at the room temperature in dark. After three times washing with PBS, cells were resuspended in PBS at a concentration of 1 × 10^6^ cells/ml. A total of 1 × 10^5^ cells were collected on a FACSCalibur (BD Bioscience, United States) and analyzed using the FlowJo software. Three replicates were included for each treatment.

### Plasmid Construction and Transfection

The CD163 genes were cloned into pcDNA3.1 plasmid containing Myc-His tag by Sanggon Biotech Corporation. pcDNA3.1-Myc plasmid and pcDNA3.1-CD163-Myc plasmid were transfected into Marc-145 cells using Lipofectamine 3000 (Invitrogen, United States) for 24 h, according to the manufacturer’s instructions. After transfection, cells were mock treated or treated with 100 ng/mL LPS for 6 h, and then infected with CHR6 (MOI = 1) for another 24 h. Cells were harvested for qRT-PCR and western blot.

### Detection of Soluble CD163 (sCD163)

After LPS treatment, the cell supernatants were collected and measured the concentration of sCD163 using porcine CD163 ELISA kit (Laibio, China) according to the manufacturer’s instructions. Three replicates were included for each treatment.

### Statistical Analysis

All experiments were performed with at least three independent replicates. Statistical analysis was performed using SPSS 17.0 and GraphPad Prism 5.0. Data presented as mean ± standard errors of the means (SEM). Student’s *t*-test and one-way ANOVA were used to analyze the data. *P* < 0.05 was considered to be significant.

## Results

### LPS Inhibits PRRSV Replication

**FIGURE 1:**
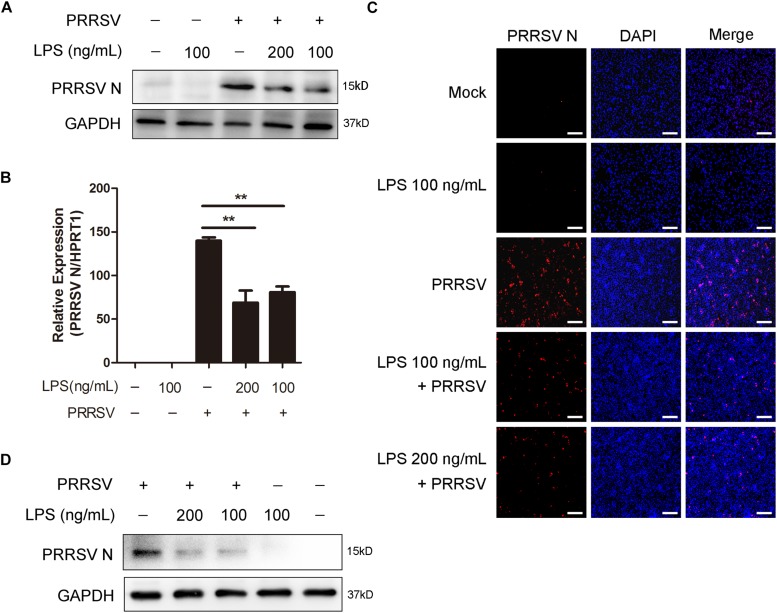
LPS restrains the replication of PRRSV. **(A–C)** PAMs were pre-treated with different concentrations of LPS (0, 100, and 200 ng/mL) for 6 h, then mock inoculated or inoculated with CHR6 at an MOI of 1 for 24 h. **(A)** Western blot was used to analysis the expression of PRRSV N. GAPDH is shown as an internal control. **(B)** Total cellular RNA was extracted and PRRSV N (ORF7) mRNA level was detected by qRT-PCR. **(C)** Immunofluorescence analysis of PRRSV N expression in PAMs. The N protein of PRRSV was immunostained using a mouse anti-N protein antibody, followed by an Alexa fluor 555-conjugated anti-mouse IgG (Red). Nuclei were counterstained with DAPI (Blue). Bar = 200 μm. **(D)** Marc-145 cells were stimulated with LPS (0, 100, and 200 ng/mL) for 6 h, and then mock infected or infected with CHR6 (MOI = 1) for 24 h. PRRSV N protein expression level was analyzed using western blot with GAPDH as an internal control. Data are representative of the results of three independent experiments (means ± SE). Significant differences compared with control group are denoted by **P < 0.01.

To explore the antiviral activity of LPS against PRRSV infection, PAMs were exposed to different concentrations of LPS (0, 100, and 200 ng/mL) for 6 h and then infected with PRRSV (MOI = 1) for another 24 h. As shown in Figures 1A,B, LPS decreased the PRRSV nucleocapsid (N) protein level and significantly reduced the mRNA level of PRRSV N. Immunofluorescence analysis also demonstrated that PRRSV N was markedly reduced following LPS treatment (Figure 1C), which indicates that LPS restrains the replication of PRRSV. To further confirm the inhibitory effect of LPS on virus infection, we conducted the same experiments in Marc-145 cells. Similarly, the expression of PRRSV N was decreased when cells were stimulated with LPS (Figure 1D). Collectively, LPS effectively restrains the replication of PRRSV in PAMs and Marc-145 cells.

### LPS Stimulation Promotes Activation of NF-κB Pathway During PRRSV Infection

**FIGURE 2:**
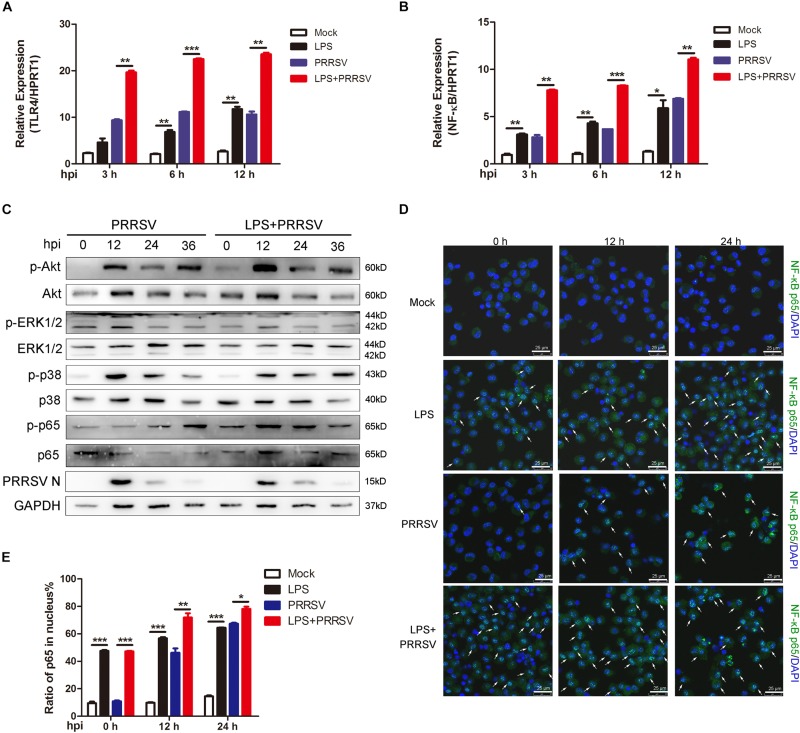
LPS promotes activation of NF-κB at the early stage of PRRSV infection. PAMs were mock treated or treated with LPS (100 ng/mL) for 6 h, and then mock infected or infected with CHR6 (MOI = 1) for various time points. **(A,B)** Total RNA was extracted at the indicated time points (3, 6, and 12 hpi) and used as template for qRT-PCR using primers specific for TLR4 **(A)** and NF-κB **(B)**. HPRT1 was used for normalization. **(C)** Phosphorylated Akt, ERK1/2, p38, p65, and N protein of PRRSV were detected using specific antibodies at 0, 12, 24, and 36 hpi. GAPDH is shown as an internal control. **(D)** Immunofluorescence analysis of p65 nuclear translocation. At 0, 12, and 24 hpi, cells were fixed and stained for NF-κB p65 followed by an Alexa fluor 488-conjugated anti-rabbit IgG (Green). Nuclei were counterstained with DAPI (Blue). White arrows represented for p65 nuclear translocation. Bar = 25 μm. **(E)** Cells treated as in **(D)** were manually quantified for p65 nuclear translocation at each time point. Data are the results of three independent experiments (means ± SE). Significant differences are denoted by *P < 0.05, **P < 0.01, and ***P < 0.001.

Previous studies show that LPS triggers innate immune response and facilitates the secretion of inflammatory cytokines (Liu et al., 2018). To investigate which signal pathway participates in LPS-induced proinflammatory response, PAMs were mock infected or infected with CHR6 (MOI = 1) in the presence or absence of LPS (100 ng/mL) for indicated time. First, we detected TLR4 expression at 3, 6, and 12 h post infection (hpi), the receptor involved in the LPS response. As shown in Figure 2A, the mRNA expression of TLR4 was significantly increased when PAMs were infected with PRRSV in the presence of LPS, compared to those treated with PRRSV alone. The activation of TLR4 triggers its downstream signaling, such as NF-κB, ERK, MAPK p38, and PI3K/Akt pathways. We found that LPS largely promoted the transcripts of NF-κB in PRRSV-infected PAMs at 3, 6, and 12 hpi, compared to PRRSV-treated cells alone (Figure 2B). The phosphorylation of Akt, ERK1/2, p38, and p65 was analyzed, which represents the activation of PI3K, ERK, MAPK p38, and NF-κB signaling, respectively. Compared with control, we found that Akt phosphorylation occurred as early as 0 hpi and was markedly enhanced at 12 hpi in PAMs co-treated with PRRSV and LPS. Moreover, p65 phosphorylation was also obviously increased in LPS-stimulated cells following PRRSV infection (Figure 2C), indicating that LPS accelerated the activation of PI3K and NF-κB signaling during PRRSV infection. Conversely, there was no observed difference in the phosphorylation of ERK1/2 and p38. It is known that the translocation of p65 into the nucleus implies the activation of NF-κB signaling. The intracellular localization of p65 was assessed by immunofluorescence assay. Consistently, upon LPS treatment prior to virus infection in PAMs, nuclear translocation of p65 was significantly increased at 12 and 24 hpi compared to cells treated with PRRSV alone (Figures 2D,E). These data demonstrate that LPS accelerates the activation of NF-κB pathway during PRRSV infection, which appears principally responsible for antiviral signaling against PRRSV.

### LPS Promotes Early Proinflammatory Response During PRRSV Infection

**FIGURE 3:**
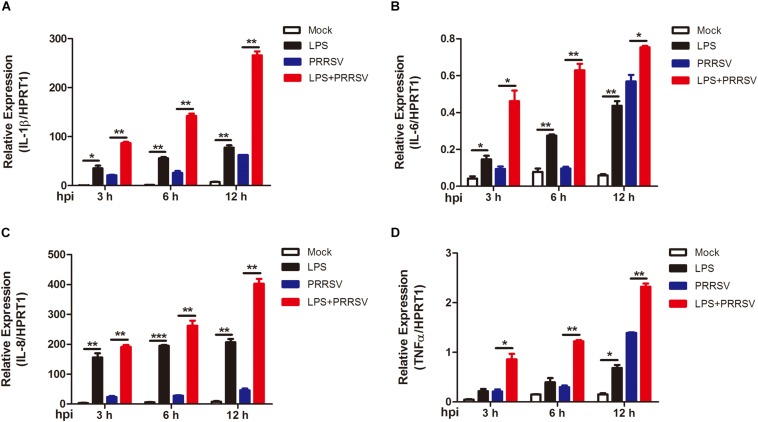
LPS induces intensive proinflammatory cytokines during PRRSV infection. PAMs were mock infected or infected with CHR6 (MOI = 1) in the presence or absence of LPS (100 ng/mL) for indicated time. Total RNA was extracted from cell lysates at 3, 6, and 12 hpi. The relative expression of IL-1β **(A)**, IL-6 **(B)**, IL-8 **(C),** and TNF-α **(D)** was analyzed using qRT-PCR. Data are normalized to HPRT1 in each individual sample. Data are the results of three independent experiments (means ± SE). Significant differences are denoted by *P < 0.05, **P < 0.01, and ***P < 0.001.

PRRSV induces NF-κB activation and leads to the release of inflammatory mediator in the late phase of PRRSV infection (Sun et al., 2012; Zhu et al., 2012). To determine whether LPS regulates the time course of proinflammatory response during PRRSV infection, PAMs were mock infected or infected with PRRSV (MOI = 1) in the presence or absence of LPS (100 ng/mL) at 3, 6, and 12 hpi. qRT-PCR was conducted to detect the expression of proinflammatory cytokines IL-1β, IL-6, IL-8, and TNF-α. As shown in Figure 3, the expression of IL-1β, IL-6, IL-8, and TNF-α was slightly increased at 3 and 6 hpi, but at a moderate level, with PRRSV infection alone. However, the mRNA expression of these cytokines was significantly elevated and to a greater magnitude in LPS- and PRRSV- cotreated PAMs compared to those treated with PRRSV alone. LPS induced higher proinflammatory cytokines expression at every time point post PRRSV infection than those without LPS treatment, which is consistent with the data observed in activation of NF-κB by LPS. These results suggest that LPS accelerates the proinflammatory response at the early stage of PRRSV infection. Taken together, LPS can induce and potentiate proinflammatory response via NF-κB signaling during PRRSV infection.

### LPS Stimulation Accelerates Activation of ADAM17

**FIGURE 4:**
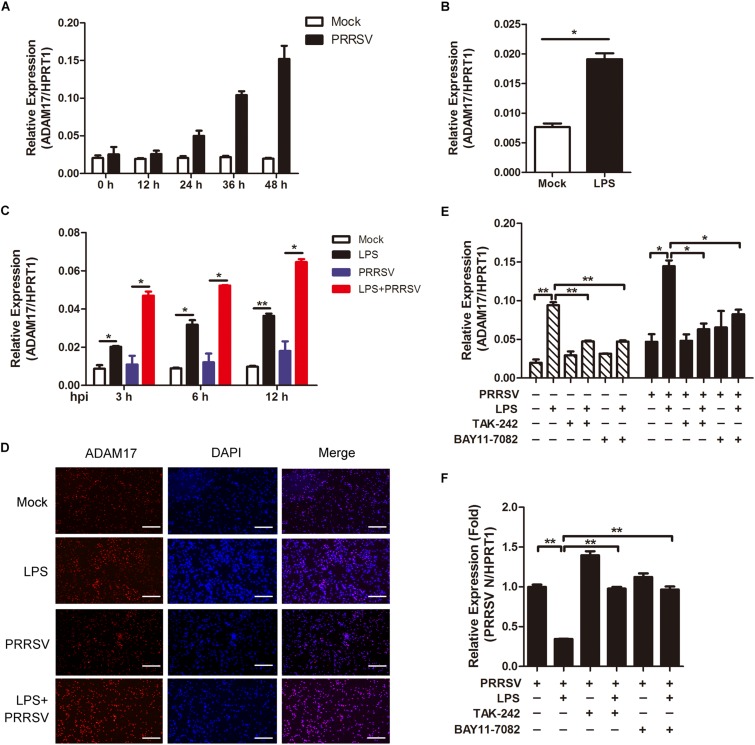
LPS stimulation improves the expression of ADAM17 following PRRSV infection. **(A)** Time course of ADAM17 expression. PAMs were mock infected or infected with CHR6 (MOI = 1) for indicated time points. Total RNA was extracted to detect the relative expression of ADAM17 mRNA using qRT-PCR. Data are normalized to HPRT1 in each individual sample. **(B)** PAMs were mock treated or treated with LPS (100 ng/mL) for 6 h. qRT-PCR was used to measure the mRNA expression of ADAM17. **(C)** PAMs were mock stimulated or stimulated with LPS (100 ng/mL) for 6 h, and then mock infected or infected with CHR6 (MOI = 1) for various time points. The mRNA expression of ADAM17 was measured at 3, 6, and 12 hpi. Data are normalized to HPRT1 in each individual sample. **(D)** Immunofluorescence analysis of ADAM17 expression. PAMs were mock infected or infected with CHR6 (MOI = 1) in the presence or absence of LPS (100 ng/mL) for 24 h. Nuclei were counterstained with DAPI (Blue). Bar = 200 μm. **(E,F)** PAMs were mock treated or treated with LPS (100 ng/mL) in the presence or absence of TAK-242 (TLR4 inhibitor, 10 μM) or BAY11-7082 (NF-κB inhibitor, 5 μM) for 6 h, then mock infected or infected with CHR6 (MOI = 1) for 24 h. The transcripts of ADAM17 **(E)** and PRRSV N **(F)** were detected using qRT-PCR. Data are normalized to HPRT1 in each individual sample. Relative expression (fold) in comparison with PRRSV infected cells alone (set up as 1) is shown. Data are the results of three independent experiments (means ± SE). Significant differences are denoted by *P < 0.05 and **P < 0.01.

ADAM17 is a transmembrane metalloproteinase mediating the shedding of the extracellular domain of several transmembrane proteins. In inflammatory condition, ADAM17 is activated with proteolytic cleavage activity (Menghini et al., 2013). We would like to investigate whether ADAM17 is activated in LPS-induced pro inflammatory condition. First, we tried to identify the tendency of ADAM17 expression post PRRSV infection. As shown in Figure 4A, the mRNA expression of ADAM17 was gradually increased at the periods of infection. To investigate whether LPS has an influence on ADAM17 expression, we used LPS (100 ng/mL) to stimulate PAMs for 6 h, prior to PRRSV challenge (MOI = 1), and detected the transcript of ADAM17 at different time points (3, 6, and 12 h). We found that the expression of ADAM17 was enhanced after LPS stimulation for 6 h (Figure 4B). Compared with virus infection alone, PAMs co-treated with LPS and PRRSV showed higher transcription level of ADAM17 at every time point of infection (Figure 4C). Consistently, the protein expression of ADAM17 is much more intense in PAMs stimulated with LPS than mock-treated cells using immunofluorescence analysis. The same results were obtained even when PAMs were challenged with PRRSV for 24 h (Figure 4D). To further demonstrate LPS accelerates activation of ADAM17 via TLR4-NF-κB signaling, cells were co-treated with LPS and TLR4 or NF-κB inhibitor (TAK-242 and BAY11-7082). The mRNA expression of ADAM17 was upregulated when cells were treated with LPS alone. However, the LPS-induced upregulation of ADAM17 was significantly decreased when cells were exposed to TLR4 or NF-κB inhibitor in the presence or absence of PRRSV (Figure 4E). Simultaneously, the expression of PRRSV N was rescued when cells were co-treated with LPS and inhibitor in comparison with LPS treated alone (Figure 4F). Collectively, LPS stimulation intensifies proinflammatory response via TLR4-NF-κB signaling, thus reinforcing the expression of ADAM17 at the early PRRSV infection.

### LPS Downregulates CD163 Expression and Promotes the Cleavage of Membrane CD163

**FIGURE 5:**
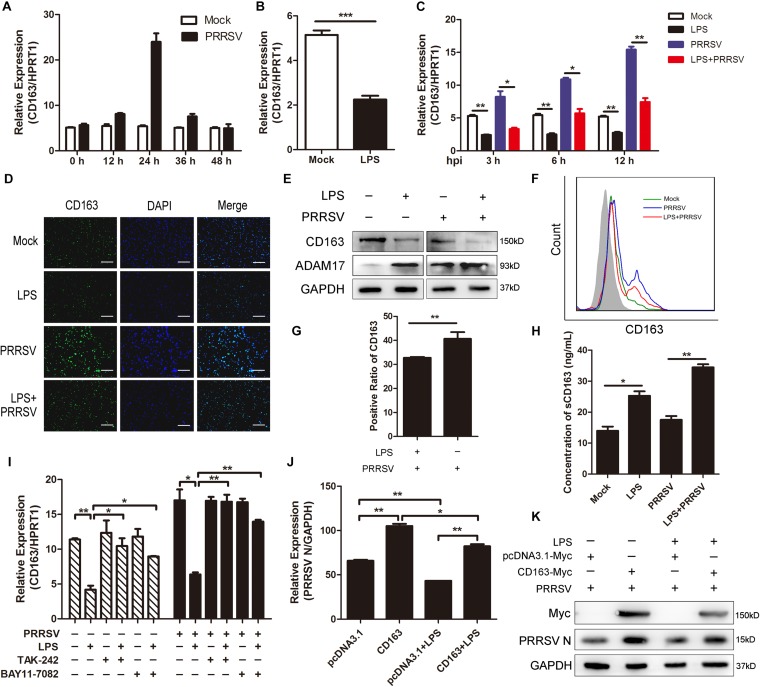
LPS downregulates CD163 expression. **(A)** Time course of CD163 expression. PAMs were mock infected or infected with CHR6 (MOI = 1) for indicated time points. qRT-PCR was used to detect the relative expression of CD163 mRNA. Data are normalized to HPRT1 in each individual sample. **(B)** PAMs were mock stimulated or stimulated with LPS (100 ng/mL) for 6 h. The mRNA of CD163 was measured using qRT-PCR. **(C)** PAMs were mock treated or treated with LPS (100 ng/mL) for 6 h, and then mock infected or infected with CHR6 (MOI = 1) for indicated time points (3, 6, and 12 hpi). The transcripts of CD163 were measured using qRT-PCR. HPRT1 was used for normalization. **(D–H)** PAMs were mock infected or infected with CHR6 (MOI = 1) in the presence or absence of LPS (100 ng/mL) for 24 h. **(D)** Immunofluorescence analysis of CD163 expression. Nuclei were counterstained with DAPI (Blue). Bar = 200 μm. **(E)** The protein expression of CD163 and ADAM17 was detected by western blot using indicated antibodies. GAPDH is shown as an internal control. **(F)** The expression of CD163 on cell surface was analyzed by flow cytometry using an anti-pig CD163-FITC antibody. **(G)** Positive cell ratio of cell surface CD163 based on analysis conditions in **(F)**. **(H)** Cell supernatants were collected and used to measure the concentration of sCD163. **(I)** PAMs were mock treated or treated with LPS (100 ng/mL) in the presence or absence of TAK-242 (10 μM) or BAY11-7082 (5 μM) for 6 h, then mock infected or infected with CHR6 (MOI = 1) for 24 h. The mRNA expression of CD163 was detected using qRT-PCR. **(J,K)** pcDNA3.1-Myc plasmid and pcDNA3.1-CD163-Myc plasmid were transfected into Marc-145 cells for 24 h, respectively. After transfection, cells were infected with CHR6 (MOI = 1) in the presence or absence of LPS (100 ng/mL) for another 24 h. PRRSV N mRNA level was detected using qRT-PCR **(J)**. Data are normalized to GAPDH in each individual sample. Western blot was used to detect the protein level of PRRSV N and Myc-labeled CD163 **(K)**. GAPDH is shown as an internal control. Data are the results of three independent experiments (means ± SE). Significant differences are denoted by *P < 0.05, **P < 0.01, and ***P < 0.001.

In the above data, LPS promoted the release of proinflammatory cytokines and ADAM17 expression. It was reported that many inflammatory factors have influence on the expression of CD163 and that ADAM17 is responsible for cleavage of the membrane-proximal region of CD163 (Etzerodt and Moestrup, 2013). Therefore, we investigated whether CD163 expression is regulated by LPS stimulation. We first detected the mRNA expression of CD163 at different time points of infection and found that CD163 expression gradually rose to the highest at 24 hpi and then fell (Figure 5A). Next, we used LPS (100 ng/mL) to stimulate PAMs for 6 h and found that the expression of CD163 was significantly decreased compared to control group (Figure 5B). As expected, CD163 mRNA expression was downregulated at various time points post PRRSV infection (MOI = 1) following LPS treatment (100 ng/mL) (Figure 5C). Fluorescence intensity of CD163 was weakened when cells were exposed to LPS in the presence of PRRSV infection, indicating that LPS downregulates the expression of CD163 (Figure 5D). The protein expression of CD163 and ADAM17 was measured by western blot following LPS treatment. Consistently, compared with mock-treated cells, the protein level of ADAM17 was remarkably increased while CD163 was observably reduced in LPS-stimulated PAMs. The same results were obtained when cells were challenged with PRRSV for another 24 h (Figure 5E). To further identify the cleavage of membrane CD163 mediated by ADAM17 upon LPS treatment, we detected the expression of membrane CD163 using flow cytometry. As expected, compared with PAMs with PRRSV treatment alone, the expression of membrane CD163 was decreased in cells co-treated with LPS and PRRSV (Figure 5F). The positive cell ratio of CD163 in LPS-treated PAMs was lower than cells without LPS stimulation following PRRSV infection for 24 h (Figure 5G). We also detected the concentration of sCD163 in cell supernatants, cells treated with LPS produced more sCD163 than cells without LPS treatment (Figure 5H). To demonstrate the role of CD163 in the TLR4 mediated inhibition of the viral infectivity, we used LPS and TLR4 or NF-κB inhibitor to co-treat PAMs. The mRNA expression of CD163 was decreased when cells were treated with LPS alone. However, the LPS-mediated inhibition of CD163 was significantly rescued when TLR4 or NF-κB signaling was blocked in the presence or absence of PRRSV (Figure 5I). Accordingly, the expression of PRRSV N was increased when cells were co-treated with LPS and inhibitor in comparison to LPS treated alone (Figure 4F). Furthermore, we used pcDNA3.1-Myc and pcDNA3.1-CD163-Myc plasmid to transfect Marc-145 cells for 24 h, respectively. After transfection, LPS treatment was carried out. We found LPS inhibited PRRSV N expression both in mock transfected and CD163-overexpressing Marc-145 cells. LPS stimulated CD163-overexpressing Marc-145 cells showed a higher viral replication in comparison to the mock transfected Marc-145 cells treated with LPS (Figures 5J,K). To conclude, the stronger proinflammatory response induced by LPS results in activation of ADAM17 and a decrease of CD163 expression, thus suppressing the replication of PRRSV.

## Discussion

The inflammatory process is precisely regulated, which contributes to induction of protective innate and adaptive immune response. Inflammatory response is triggered at the time that bacteria and viruses invade *in vivo* or *in vitro*. Signals related to inflammatory process are activated and regulate inflammation, including proinflammatory or anti-inflammatory reaction. Among these signals, NF-κB signaling pathway plays a crucial role in the transcription of inflammatory or immunomodulatory genes (Hao et al., 2015). The activation of NF-κB signaling induces intensive expression of proinflammatory cytokines and type I interferon, which shows powerful protection against foreign pathogens. TLR4 is identified as a receptor that responds to LPS, which tightly associates with its downstream NF-κB signaling. Although TLR4-MD2-LPS complex initially triggers the early-phase activation of NF-κB in a MyD88-dependent manner while the activation of late-phase NF-κB in a TRIF-dependent manner, the induction of inflammatory cytokines is caused by the activation of TLR4-NF-κB in both two pathways (Kawai and Akira, 2010). It is reported that PRRSV arouses NF-κB activation and leads to the release of several cytokines (Lee and Kleiboeker, 2005; Sun et al., 2012; Ke et al., 2019). Another study demonstrates TLR4/MyD88/NF-κB signaling pathway is involved in IL-1β production during PRRSV infection (Bi et al., 2014). In our study, it was interesting to find that the activation of NF-κB was inhibited at the early stage of PRRSV infection, and PRRSV triggered the activation of NF-κB until middle and late stages of infection in PAMs. Consistently, there was a delayed NF-κB-mediated proinflammatory response during PRRSV infection. When PAMs were stimulated with LPS prior to PRRSV challenge, we found that the stronger activation of TLR4-NF-κB pathway and its signal molecules compared to cells with PRRSV treated alone (Figure 2). Likewise, proinflammatory cytokines were largely induced in LPS-treated PAMs as compared with control during PRRSV infection (Figure 3). Collectively, LPS significantly promotes the early activation of NF-κB and potentiates the expression of proinflammatory cytokines. A certain amount of TLR4-NF-κB activation benefits to the host by improving a protective immune response against viral infection (Olejnik et al., 2018). Moreover, TLR4 also triggers its downstream signaling, such as ERK, MAPK p38 pathways, which are inflammation-related signaling. As our results shown, PRRSV could activate ERK1/2 and p38 signaling, but there was no obvious difference in the phosphorylation of ERK1/2 and p38 when cells were treated with additional LPS (Figure 2C). The possible reason is that phosphorylation of ERK1/2 and p38 remains plateau upon PRRSV infection, thus the effect is mostly associated to PRRSV with no additional impact after LPS treatment.

Macrophages are polarized toward two phenotypes in response to different stimulus. IFN-γ and LPS polarizes macrophages toward M1 phenotype, which shows intensive proinflammatory response to kill intracellular pathogens. On the contrary, macrophages are polarized to M2 phenotype following IL-4, IL-10, or IL-13 stimulation, which triggers anti-inflammatory response (Gordon, 2003). Previous studies have shown that PAMs polarized toward M1 phenotype significantly inhibit PRRSV replication (Wang et al., 2017). In our work, LPS-treated PAMs appeared enhanced expression of proinflammatory cytokines, including IL-1β, IL-6, IL-8, and TNF-α, which are prototypical M1 macrophage markers (Figure 3). Therefore, PAMs polarization and proinflammatory cytokines upregulation may be an explanation of the inhibition of PRRSV by LPS.

**FIGURE 6:**
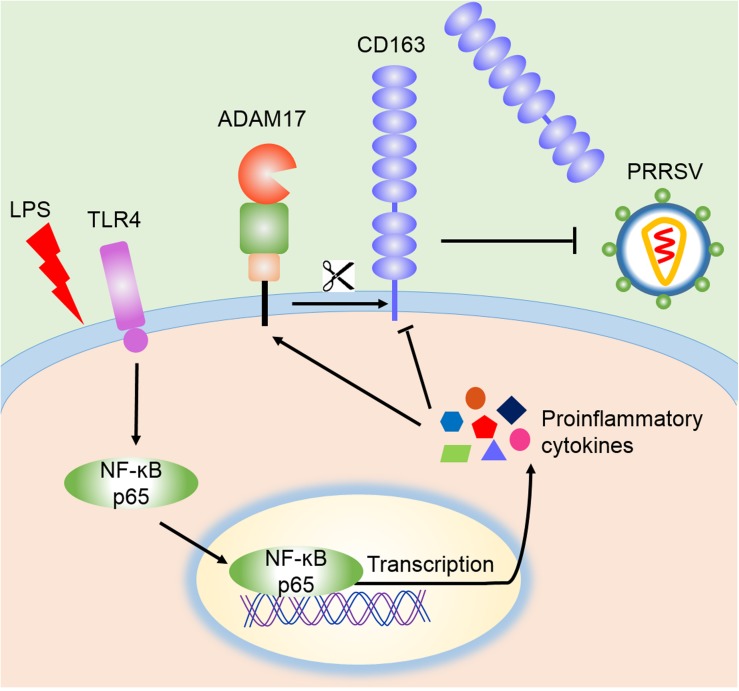
Schematic model of the inhibition of PRRSV by LPS. According to our data, LPS stimulation leads to the activation of TLR4 and NF-κB signaling, which facilitates the transcription of proinflammatory cytokines and potentiates proinflammatory response. These proinflammatory factors activate ADAM17 to cleave CD163, resulting in the shedding of membrane CD163, which ultimately leads to the inhibition of PRRSV.

CD163 is not only a key receptor for PRRSV infection, but also participates in inflammatory process (Van Gorp et al., 2010; Welch and Calvert, 2010). Anti-inflammatory mediators up-regulate the expression of CD163, while down-regulation of CD163 is driven by endogenous or exogenous proinflammatory factors, including IL-1α, IL-1β, IL-4, IL-8, TNF-α, and LPS. Activation of TLR4 also leads to a reduction of CD163 expression (Kowal et al., 2011). Membrane CD163 is cleaved by ADAM17, a metalloprotease, which regulates PRRSV entry process (Guo et al., 2014). In our research, LPS stimulation triggered the activation of TLR4-NF-κB pathway and early proinflammatory response, which reduced the expression of CD163 and increased the cleavage of membrane CD163 mediated by enhanced ADAM17 and eventually resulted in the inhibition of PRRSV (Figure 6). To demonstrate the role of CD163 in the TLR4 mediated inhibition of the viral infectivity, we used specific TLR4 or NF-κB inhibitor to block LPS response, and found that ADAM17 was decreased while CD163 and PRRSV N were significantly increased compared to LPS treated alone. Meanwhile, LPS stimulated CD163-overexpressing Marc-145 cells showed a higher viral replication in comparison to the mock transfected Marc-145 cells. These data indicate that the antiviral effect of the TLR4 stimulation is attributed to the down-regulation of CD163.

Our present study revealed that LPS stimulation enhances proinflammatory response via TLR4-NF-κB pathway, leading to an increase of ADAM17 and a reduction of CD163, which results in the inhibition of PRRSV infection. These findings provide important clues for the mechanism that inflammation regulates viral infection, which will help us to find better antiviral therapy.

## Data Availability Statement

All datasets generated for this study are included in the article/supplementary material.

## Ethics Statement

The animal study was reviewed and approved by the Institutional Animal Care and Use Committee of Sun Yat-sen University.

## Author Contributions

ZZ and CG conceived and designed the study. ZZ and HZ performed the experiments, analyzed the data, and drafted the manuscript. XZ, SH, WD, XW, and YC coordinated the study. ZZ, XL, and CG contributed to the interpretation of the data and took part in the critical revision of the manuscript. All authors have read and approved the final manuscript.

## Conflict of Interest

The authors declare that the research was conducted in the absence of any commercial or financial relationships that could be construed as a potential conflict of interest.
